# Catechins induced acute promyelocytic leukemia cell apoptosis and triggered PML-RARα oncoprotein degradation

**DOI:** 10.1186/s13045-014-0075-3

**Published:** 2014-10-01

**Authors:** Li Zhang, Qiu-Sheng Chen, Peng-Peng Xu, Ying Qian, Ai-Hua Wang, Dan Xiao, Yan Zhao, Yan Sheng, Xiang-Qin Wen, Wei-Li Zhao

**Affiliations:** State Key Laboratory of Medical Genomics, Shanghai Institute of Hematology, Department of Hematology, Rui Jin Hospital, Shanghai Jiao Tong University School of Medicine, 197 Rui Jin Er Road, Shanghai, 200025 China; Pôle de Recherches Sino-Français en Science du Vivant et Génomique, Laboratory of Molecular Pathology, Shanghai, China

**Keywords:** Catechins, Acute promyelocytic leukemia, Apoptosis, PML-RARα oncoprotein

## Abstract

**Background:**

It has recently been reported that the extracts of green tea polyphenol have cancer preventive effects. In this study, we investigated the effect of the natural composition from green tea leaves Catechins on acute promyelocytic leukemia (APL).

**Methods:**

In vitro, APL cell lines NB4, retinoic acid-resistant NB4-R1 and NB4-R2 were treated with different concentrations of Catechins. Cell viability and cell apoptosis were analyzed using MTT assay and flow cytometric assay, respectively. Expression of proteins related to apoptosis and PML-RARα oncoprotein were assessed by Western blot. In vivo anti-tumor activity of Catechins was examined in nude mice xenografted with NB4 cells and in situ cell apoptosis was detected by terminal deoxytransferase-catalyzed DNA nick-end labeling assay.

**Results:**

Catechins at micromolar concentration levels significantly inhibited APL cell proliferation and induced cell apoptosis, in association with mitochondria damage, ROS production and caspase activation. The anti-apoptotic Bcl-2 family member Bcl-xL was down regulated, with pro-apoptotic member Bax remaining unchanged. Moreover, Catechins induced the degradation of PML-RARα oncoprotein. Catechins-mediated apoptotic effect was also observed in primary APL cells without affecting normal hematopoietic progenitor cells. In the murine xenograft model, Catechins remarkably inhibited tumor growth and induced in situ leukemic cell apoptosis.

**Conclusions:**

Catechins might be a potential candidate for APL treatment by activating intrinsic apoptotic pathway and targeting PML-RARα oncoprotein.

## Background

Acute promyelocytic leukemia (APL) accounts for approximately 10% of all acute myeloid leukemias and is characterized by a specific chromosomal translocation t(15;17), resulting in the fusion of promyelocytic leukemia (PML) gene to retinoic acid receptor (RARα) gene. The expression of PML-RARα chimeric protein plays a central role in leukemogenesis, including arrest of differentiation and deregulation of apoptosis [1,2]. The currently used agents all-trans retinoic acid (ATRA) and arsenic trioxide (As_2_O_3_) directly target PML-RARα oncoprotein and dramatically improve the clinical outcome of APL patients [3–13]. This greatly encourages further discovery of potential molecular target-based agents, particularly nature products, on APL treatment.

Epidemiologic studies have already shown that green tea consumption is beneficial to health and can reduce the incidence of cancer [14]. Recently, green tea products have attracted more attention because of their anti-cancer effects revealed in experimental tumor models [15–32]. Catechins is the main component extracted from the green tea leaves, including epipallocatechin gallate (EGCG), epicatechin gallate (ECG), epigallocatechin (EGC) and epicatechin (EC) etc. [33]. Catechins prove to be inexpensive, safe, and can be administrated orally. Therefore, whether Catechins possesses anti-leukemia capability is of great interest to leukemia treatment.

In this study, we assessed the effect of Catechins on both retinoic acid (RA)-sensitive and -resistant APL cell lines [34–36], as well as on primary APL cells and on a murine xenograft APL model. The Catechins-induced apoptosis of APL cells and expressions of related proteins (Bcl-2, Bcl-xL, Bax and PML-RARα) were also investigated to explore possible molecular mechanism.

## Results

### Catechins inhibited cell growth and induced cell apoptosis in human APL cell lines

**Figure 1:**
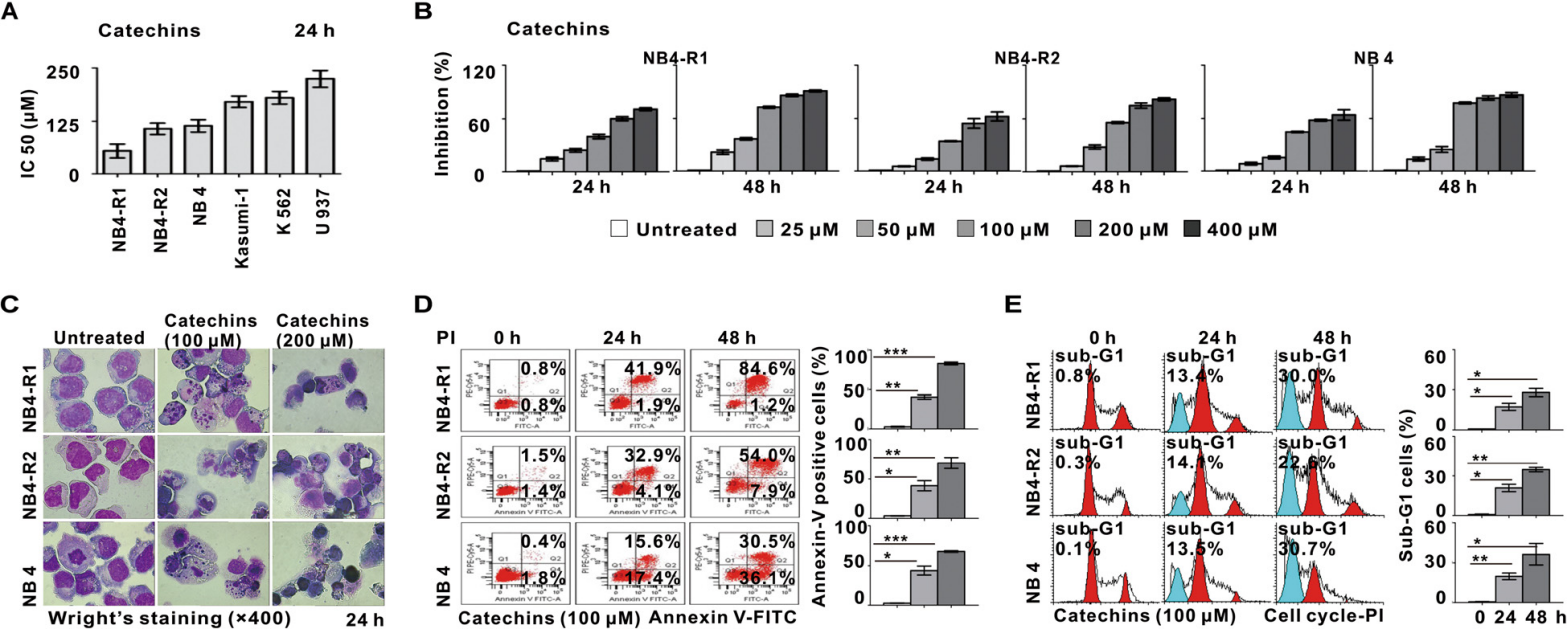
**The effect of Catechins treatment on growth and apoptosis of human leukemia cell lines. (A)** IC50 results obtained from MTT assay in leukemia cell lines treated with Catechins at 24 h. The IC50 values of NB4-R1, NB4-R2 and NB4 cells were below 125 μM. **(B)** The growth inhibition of NB4-R1, NB4-R2 and NB4 cells treated with Catechins for 24 and 48 h. Reduced cell viability were detected in APL cell lines from 50 μM Catechins. **(C)** Characteristic apoptotic cells were present in NB4-R1, NB4-R2 and NB4 cells treated with 100 μM or 200 μM Catechins for 24 h. **(D)** Detection of apoptotic cells by Annexin V-FITC/PI double staining in NB4-R1, NB4-R2 and NB4 cells treated with 100 μM Catechins for 24 and 48 h. Catechins treatment increased the percentages of Annexin-V+/PI- cells (lower right quadrant) and Annexin-V+/PI + cells (upper right quadrant). **(E)** Contribution of nuclear DNA content in NB4-R1, NB4-R2 and NB4 cells treated with 100 μM Catechins for 24 h and 48 h. Significantly increased sub-G1 cells were observed. *P < 0.05, **P < 0.01, ***P < 0.001 comparing with the untreated cells.

Using MTT assay, we determined the effect of Catechins on various human leukemia cell lines. The IC50 value (median inhibitory concentration) of Catechins in these leukemia cells were calculated after 24 hours of treatment. Catechins exerted substantial growth inhibition in APL cell lines (NB4-R1, NB4-R2 and NB4), Kasumi-1 cells and K562 cells (Figure 1A). However, the sensitivity of U937 cells to Catechins was relatively lower, with IC50 higher than 200 μM.

The response curves of NB4-R1, NB4-R2 and NB4 to Catechins were shown in Figure 1B. Catechins inhibited cell growth in a time- and dose-dependent manner. To confirm whether the growth inhibition of Catechins was caused by apoptosis, cell morphology and AnnexinV-FITC/PI double staining were performed. Morphologically, cell apoptosis was observed at 24 hours of treatment with Catechins, showing characteristic changes, such as chromatin condensation, nuclear fragmentation, and formation of apoptotic bodies (Figure 1C). The percentage of Annexin V-positive cells, reflecting those undergoing apoptosis, was gradually increased during treatment (Figure 1D). Cell cycle analysis also revealed a time-dependent elevation of sub-G1 cell content, consistent with Catechins-induced APL cell apoptosis (Figure 1E).

### Catechins-induced apoptosis was associated with mitochondria damage, ROS production and caspase activation

**Figure 2:**
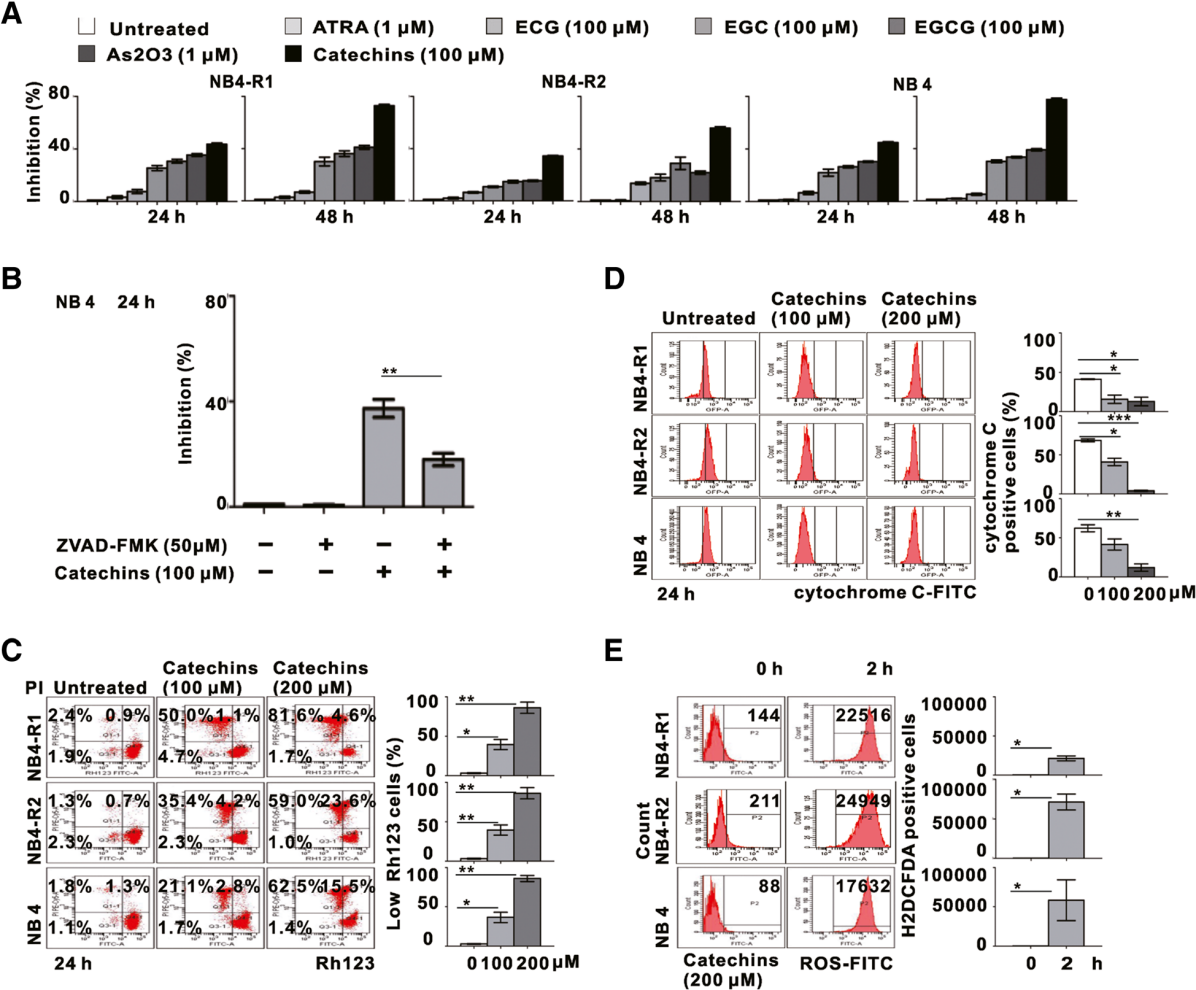
**Catechins induced mitochondrial trans-membrane potential collapse, cytochrome C loss and ROS production in NB4 cells. (A)** The inhibitory effect of NB4-R1, NB4-R2 and NB4 cells in response to ATRA (1 μM), As_2_O_3_ (1 μM), EGCG (100 μM), ECG (100 μM), EGC (100 μM) and Catechins (100 μM) treatment for 24 and 48 h. **(B)** Growth inhibition of NB4 cells was significantly abrogated by pan-caspase inhibitor ZVAD-FMK (50 μM). **P < 0.01 comparing with the Catechins group. **(C)** The mitochrondrial trans-membrane potential was decreased in NB4-R1, NB4-R2 and NB4 cells treated with 100 μM and 200 μM Catechins for 24 h. The numbers below the scatter plots represented the percentage of Rh123 low cells. **(D)** The mitochondrial cytochrome c was accordingly decreased in NB4-R1, NB4-R2 and NB4 cells treated with 100 μM and 200 μM Catechins for 24 h. **(E)** ROS generation by Catechins in NB4-R1, NB4-R2 and NB4 cells. DCF-derived fluorescence in untreated and in those treated for 2 h with 200 μM Catechins was shown. *P < 0.05, **P < 0.01, ***P < 0.001 comparing with the untreated cells.

The anti-leukemia effect of APL cell lines treated by ATRA (1 μM), As_2_O_3_ (1 μM), EGCG (100 μM), ECG (100 μM), EGC (100 μM) and Catechins (100 μM) were further compared. As shown in Figure 2A, Catechins, more effiently than its main active components, exhibited a significant growth inhibitory effect as observed in the As_2_O_3_ group, indicating that Catechins was equally effective as As_2_O_3_.

NB4 cells were then treated with Catechins, either alone or combined with pan-caspase inhibitor ZVAD-FMK. Catechins-induced cell growth inhibition could be significantly abrogated by ZVAD-FMK treatment, referring Catechins as an apoptotic-dependent cell death inducer (Figure 2B). Marked dissipation of mitochondrial trans-membrane potential (Δψm) (Figure 2C) and subsequent decreased mitochondrial cytochrome c (Figure 2D) were observed in NB4-R1, NB4-R2 and NB4 cells treated with Catechins in a dose-dependent manner.

To investigate whether ROS level was also affected by Catechins, we also used flow cytometric analysis with a cell-permeable dye, H_2_DCFDA, which is specifically cleaved to emit a fluorescence wave length in the presence of ROS. Treatment with 200 μM Catechins for 2 hours resulted in a significant elevation of intracellular ROS in NB4-R1, NB4-R2 and NB4 cells (Figure 2E).

### Catechins acted on intrinsic apoptotic pathway through downregulation of Bcl-xL and induced apoptosis-independent degradation of PML-RARα oncoprotein

**Figure 3:**
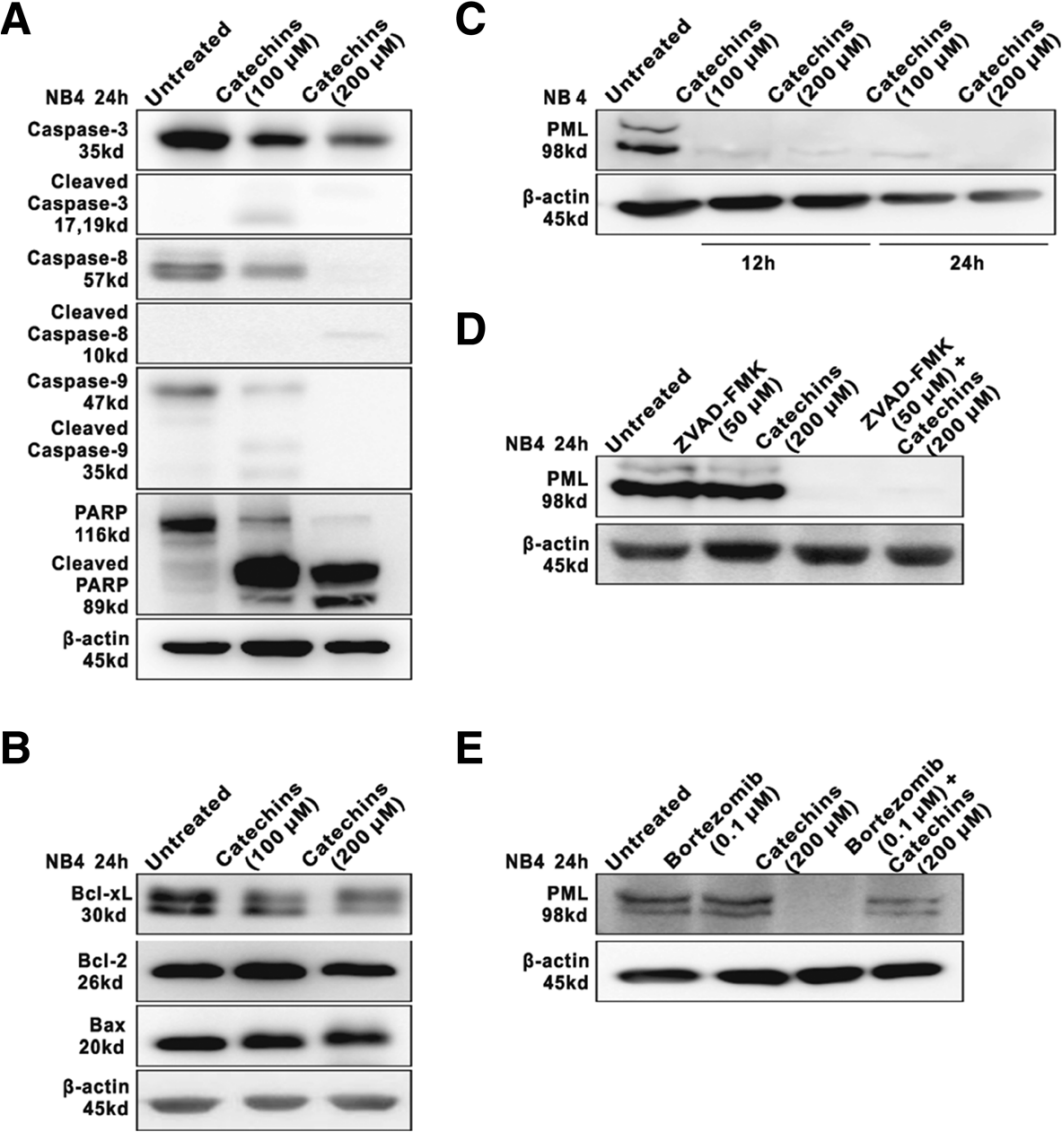
**Catechins induced caspase activation, Bcl-xL downregulation, and the degradation of PML-RARα fusion protein in NB4 cells. (A)** Western blot analysis of caspase-3, −8, −9, and PARP in NB4 cells treated with 100 μM and 200 μM Catechins for 24 h. Caspase-3 was activated with caspase-8 and caspase-9 and PARP cleaved. **(B)** The expression of Bcl-2, Bcl-xL and Bax protein were determined by Western blot. Bcl-xL expression was reduced in NB4 cells treated with 100 μM and 200 μM Catechins for 24 h. **(C)** PML-RARα was decreased upon treatment with 100 μM and 200 μM Catechins at 12 h and 24 h. **(D and E)** PML-RARα was not affected by pan-caspase inhibitor ZVAD-FMK (50 μM), but partially blocked by proteasome inhibitor bortezomib (0.1 μM). Three independent experiments were performed and representative results were shown.

In parallel to Δψm loss, western blot analysis showed that Catechins significantly induced activation of caspase-3, −8 and −9 in NB4 cells. Correspondingly, PARP was cleaved to an 89-kDa fragment by Catechins treatment at 24 hours (Figure 3A). These data suggested that Catechins induced cell apoptosis through an intrinsic mitochondrial pathway and was dependent on caspase activation.

Mitochondrial membrane permeability is directly controlled by Bcl-2 family proteins, which are the central regulators of caspase activation. To determine whether Catechins impair the mitochondria through affecting these Bcl-2 family members, the expression of anti-apoptotic factor Bcl-2 and Bcl-xL, as well as pro-apoptotic factor Bax were investigated in NB4 cells at 24 hours of incubation with Catechins. Bcl-xL expression were decreased, while no significant change was detected on Bcl-2 and Bax expression (Figure 3B).

Western blot analysis confirmed that untreated NB4 cells expressed the PML-RARα oncoprotein. Catechins treatment induced the degradation of PML-RARα at 12 and 24 hours (Figure 3C). Independent on its apoptotic action, Catechins-mediated degradation of PML-RARα oncoprotein was not affected by co-treatment with pan-caspase inhibitor ZVAD-FMK (Figure 3D). However, the degradation process could be, at least partially, blocked by proteasome inhibitor bortezomib, indicating that it was dependent on proteasome pathway (Figure 3E).

### Catechins induced apoptosis of leukemia cells from t(15;17) APL patients and did not affect the proliferation capacity of normal hematopoietic progenitor cells

**Figure 4:**
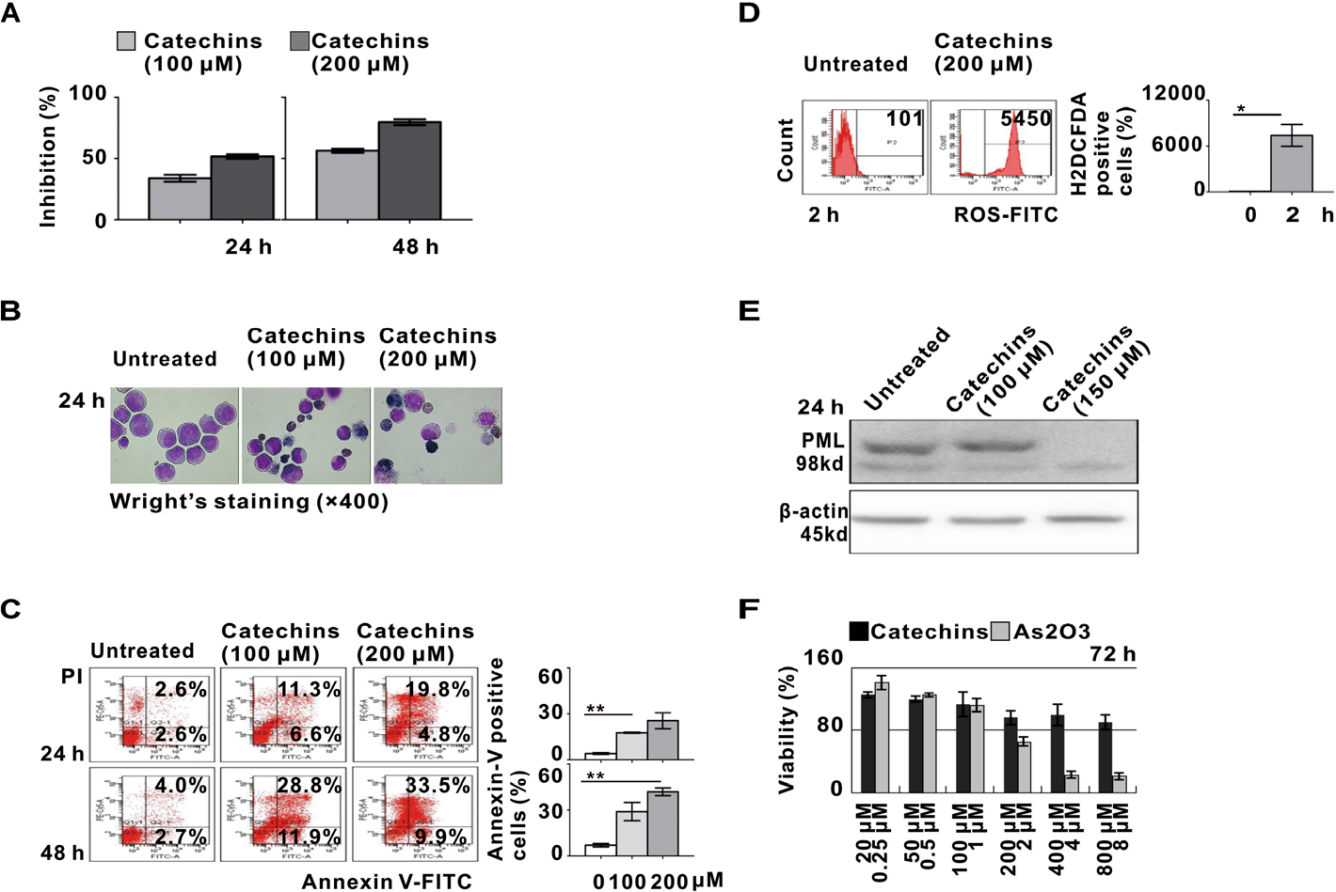
**Catechins induced apoptosis in primary APL cells without affecting the proliferation capacity of normal hematopoietic progenitor cells. (A)** Catechins inhibited growth of primary leukemia cells from patients with APL by MTT assay. Cells were treated with 100 μM and 200 μM Catechins for 24 h and 48 h. **(B)** Catechins induced apoptosis of primary leukemia cells from three patients by morphological study. Cells were treated with 100 μM and 200 μM Catechins for 24 h. **(C)** Apoptosis was evaluated by Annexin V/PI double staining and showed the fold-increase of apoptotic cells in all three cases. Cells were treated with 100 μM and 200 μM Catechins for 24 and 48 h. **P < 0.01 comparing with the untreated cells. **(D)** Intracellular levels of ROS were measured by flow cytometry in all three cases. Cells were treated with 200 μM Catechins for 2 h. *P < 0.05 comparing with the untreated cells. **(E)** The PML-RARα oncoprotein was degraded in primary leukemia cells treated with 100 μM and 150 μM Catechins for 24 h. **(F)** The proliferation of normal hematopoietic progenitor cells was not inhibited by Catechins up to 800 μM. CD34+ cells enriched from cord blood samples (n = 3) were cultured in the presence of Catechins at the indicated concentrations for 72 h. The cell viability was measured by MTT assay.

Treatment with Catechins significantly inhibited cell growth and resulted in more than 50% cell inhibition at 48 hours in primary leukemia cells from three APL patients with t(15;17) (Figure 4A). Representative morphological changes of apoptosis were present in the Catechins group (Figure 4B). Annexin V-PI double staining assay also revealed increased apoptosis in Catechins-treated cells, as compared to the untreated cells (Figure 4C). The ROS production in APL cells treated with 200 μM Catechins for 2 hours was significantly higher than that in the untreated cells (Figure 4D). Treatment with 100 μM and 150 μM Catechinsafter 24 hours significantly induced the degradation of PML-RARα oncoprotein (Figure 4E).

Of note, as determined by MTT assay, proliferation of CD34^+^ cells isolated from human cord blood was not affected even at the concentrations up to 800 μM after 72 hours of treatment, suggesting that primary APL cells responded to Catechins in a similar way as NB4 cells and Catechins exerted no severe cytotoxic effect on normal hematopoietic precursors (Figure 4F).

### Catechins inhibited tumor growth and induced in situ leukemia cell apoptosis in a murine xenograft model

**Figure 5:**
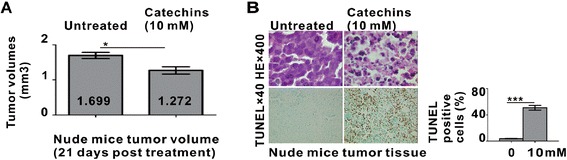
**In vivo effect of Catechins on murine models of APL. (A)** Catechins decreased the tumor volumes in a murine xenograft model. Nude mice were received 3 Gy radiation one day before subcutaneous injection with NB4 cells. The treatments began 10 days later including control diluent (n = 5) and Catechins (10 mM) (n = 5) daily for 21 days. The tumor volumes of control mice and Catechins-treated mice on Day 31 were measured. Catechins significantly reduced the xenograft tumor volumes (*P < 0.05). **(B)** Apoptotic cells detected by TdT-FragEL™ DNA Fragmentation Detection kit were calculated. A significantly increased number of apoptotic cells was observed in the Catechins group, comparing with the untreated group ***P<0.001.

We established the human APL model in nude mice using NB4 cells. NB4 cells (1 × 10^7^) were inoculated subcutaneously into the nude mice (Day 0). The latency of tumor formation at the site of injection was approximately 6–8 days. The mice were then treated with Catechins (10 mM) as the only drinking from Day 10 and tumor volume was measured daily until Day 31. Compared with the untreated group, Catechins significantly reduced the tumor size (P < 0.01) (Figure 5A). During treatment, Catechins-treated mice appeared with better condition than those of the control group. Pathologic analysis at autopsy revealed no tumor infiltration in any of the organs (data not shown).

Tumor cell apoptosis was evaluated by TUNEL assay. Comparing with the control mice, a significantly increased number of apoptotic cells was observed in the Catechins-treated mice (p < 0.001) (Figure 5B), providing in vivo evidence of Catechins-induced APL cell apoptosis.

## Discussion

Catechins is the full extracts of the natural green tea leaves. EGCG is the main component of Catechins and has been shown with anti-tumor activities in many types of cancers [15–32]. Here we reported the first time, that Catechins possessed an anti-proliferative effect on leukemia cells, especially on t(15;17) leukemia cells. This was observed not only in well-established APL cell lines and primary tumor samples of APL patients, but also in a murine xenograft tumor model of t(15;17) leukemia. Equally effective to targeted agents commonly used in APL as As_2_O_3_, Catechins, with similar bioactivity but much less expensive than EGCG, could be a potential candidate to treat APL.

Catechins possesses the anti-leukemic activity mainly due to the induction of apoptosis, which has been demonstrated by morphological features and increase of apoptotic cells both in vitro and in vivo. Caspase-3 activation is essential for leukemia cell apoptosis. In our study, Catechins generated a cleavage of caspase-3 and subsequent cleavage of the DNA repair enzyme PARP, the hallmark of apoptosis. Bcl-xL, as an important anti-apoptotic protein of Bcl-2 family [37], was accordingly reduced by Catechins, further implying that intrinsic apoptotic pathway was involved in Catechins-induced apoptosis. This is in consistent with previous study of EGCG on many solid tumors, such as hepatocellular carcinoma, chondrosarcoma, and endometrial cancer [38–42]_._ Moreover, Catechins also changed the intracellular redox status and regulatedthe mitochondria pathway through elevating ROS, similar to EGCG [43]. The intracellular redox status, depending on ROS generation, is critical in keeping mitochondria stable. Elevation of intracellular ROS production was also shown during EGCG-induced apoptosis in tumor cell lines mentioned above [38–40]. Therefore, Catechins induced APL cell apoptosis through intrinsic apoptotic pathway via Bcl-xL downregulation and ROS induction.

Interestingly, PML-RARα oncoprotein can be directly targeted by Catechins. In hepatocellular carcinoma, EGCG lowers the expression of phosphorylated STAT3 protein and inhibits the expression of multiple genes including Bcl-xL [44]. In an apoptosis-independent manner, functional modulation of RARA and PML-RARα by the peptidyl-prolyl-isomerase Pin1, or the the mitogen-activated protein kinase, p38α correlates with stabilization/degradation of PML-RARα via the proteasome pathway [45,46]. Our data also revealed that Catechins acted on PML-RARα, at least partially, in a proteasome-dependent manner. Therefore, PML-RARα oncoprotein may also represent the target of the Catechins treatment in APL, although the precise mechanism of action in Catechins-induced PML-RARα degradation need further investigation.

Our findings not only suggested possible mechanisms of Catechins in the apoptosis-regulatory pathways in APL cells, but also provide a model for studying Catechins in cancer treatment. Since green tea extracts have already entered phase I trials in patients with solid tumors [47–49], similar clinical trials would be necessary to further evaluate the anti-leukemic effect of Catechins on acute leukemias.

## Conclusions

In summary, our study demonstrated that Catechins effectively induced apoptosis of APL cells through induction of intrinsic apoptotic pathway and degradation of PML-RARα oncoprotein. Catechins may thus be a potential apoptosis inducer and therapeutic agent for APL treatment.

## Materials and methods

### Reagents

Catechins (Pharmanex, USA, each capsule contains EGCG 95 mg, ECG 37 mg, EGC 15 mg), EGCG (Sigma-ALDRICH, E4143, C22H18O11, MW: 458.37), ECG (Shanghai Winherb Medical Technology Co., Ltd, C22H18O10, MW: 442.37) and EGC (Shanghai Winherb Medical Technology Co., Ltd, C15H14O7, MW: 306.27) were prepared at the concentration of 10 mM with RPMI 1640 medium (GIBCO-BRL, Grand Island, NY, USA). Arsenic trioxide (As_2_O_3_, Sigma-ALDRICH, A1010, MW: 197.84) was dissolved in RPMI 1640 medium as 5 mM solution. All-trans-retinoic acid (ATRA, Sigma-ALDRICH) was dissolved in ethanol as 100 μM solution. Pan-caspase inhibitor ZVAD-FMK (627610) was from Merck & Co. Inc. Bortezomib was from Millennium Pharmaceuticals (Cambridge, MA, USA). The primary antibodies against β-actin (13E5, #4970), anti-caspase-3 (8G10, #9665), anti-caspase-8 (D35G2, #4790), anti-caspase-9 Antibody (#9502), anti-PARP Antibody (#9542), anti-Bcl-2 (D55G8, #4223), anti-Bax (D2E11, #5023), anti-Bcl-xL (54H6, #2764) were from Cell Signaling (Beverly, MA, USA). Mouse monoclonal anti-PML Protein (ab57276) was from Abcam (Hongkong). The secondary antibody ImmunoPure Goat Anti-Rabbit IgG (#31460) was from Thermo Scientific (USA). Goat anti-mouse IgG (PB001) was from Shanghai Immune Biotech Ltd (ImB, CHINA). Chemiluminescence phototope-horseradish peroxidase kit (WBKLS0100) was from Millipore (Germany).

### Cell lines, cell viability and cell morphology

Human APL leukemia cell lines NB4 (retinoic acid-sensitive), NB4-R1 and NB4-R2 cells (retinoic acid-resistant) were kindly provided by Professor Michel Lanotte at Saint Louis Hospital in France. Acute myeloid leukemia cell lines Kasumi-1, K562 and U937 were available from American Type Culture Collection. Cells were cultured in RPMI 1640 medium, supplemented with 10% heat-inactivated fetal bovine serum (GIBCO-BRL), 100 U/mL penicillin and 100 mg/mL streptomycin (GIBCO-BRL), in 5%CO_2_-95% air humidified atmosphere at 37°C. Fresh leukemia cells from the bone marrow of three APL patients were enriched by Ficoll separation. The diagnosis was established on the basis of morphological examination, presence of t(15;17) by cytogenetic study and PML-RARα fusion gene by molecular analysis. CD34^+^ cells were purified from human cord blood by density gradient centrifugation. Informed consent was obtained according to institutional guidelines. Cell viability was assessed by triplicate counting of trypan blue dye-excluding cells under light microscopy. Cell morphology was evaluated by Wright’s staining of cells prepared by cytospin centrifugation.

### MTT reduction assay

A total of 5 × 10^4^ cells per well were seeded in a 96-well plate, and treated with Catechins at concentrations of 25, 50, 100, 200 or 400 μM for different time (24 h, 48 h or 72 h). After treatment, 0.1 mg MTT was added to each well. The samples were incubated for 4 hours and the absorbance (optical density, OD value) was measured at 490 nm by spectrophotometry. Calculation of the cell growth inhibition rate at different concentrations is done by comparing it against the growth rate of untreated control group. Inhibition rate = [1 - OD value of treated cell/OD value of control cell] × 100%.

### Flow cytometric assays for Annexin-V/PI, nuclear DNA contentdistribution, mitochondrial trans-membrane potentials, mitochondrial cytochrome c, and reactive oxygen species (ROS) detection

A total of 2 × 10^5^ cells was analyzed using an Annexin V-FITC/PI apoptosis detection kit II (BD Pharmingen™, Franklin Lakes, NJ, USA) according to manufacturer’s instructions. To assess the distribution of nuclear DNA content, cells were collected, washed in PBS and fixed overnight in 75% ethanol at −20°C, treated with 1% RNase A for at least 15 min at 37°C, and stained with 50 μg/ml PI. For mitochondrial trans-membrane potential assessment, 1 × 10^6^ cells were washed twice with PBS, incubated with 10 μg/mL of Rh123 for 30 min at 37°C, and stained with 50 μg/mL of PI. Mitochondrial cytochrome c was measured by FlowCellect™ cytochrome c kit (Millipore) according to manufacturer’s protocols. To measure ROS levels, 5 × 10^5^ cells were washed with RPMI1640 and incubated with 5 μM DCFH-DA for 30 min at 37°C. The fluorescent intensity was measured by flow cytometry (Becton Dickinson, San Jose, CA, USA). All experiments were performed in triplicate and data were collected, stored, and analyzed by Lysis 11 software (Becton Dickinson).

### Western blot analysis

Western blot analysis was performed according to standard protocols. Approximately 5 × 10^6^ cells were harvested and incubated in 100 μL lysis buffer [50 mM Tris (pH 7.4), 150 mM NaCl, 1% Triton X-100, 1% deoxycholate, 0.1% SDS)] to prepare total protein samples. Equal amounts of protein (20 μg) were separated by SDS-PAGE on 10% gels and transferred on to PVDF membrane (Millipore), and blocked with 5% non-fat dried milk in TBST [phosphate-buffered saline-0.05% Tween] at room temperature for 60 min. The membranes were subjected to immunoblot analyses with appropriate primary antibody followed by horseradish peroxidase-linked secondary antibody. The immunocomplexes were visualized using chemiluminescence phototype-horseradish peroxidase kit.

### Terminal deoxytransferase-catalyzed DNA nick-end labeling (TUNEL) assay

In situ tumor cell apoptosis was performed on deparaffinized 5 μm thick sections using TdT-FragEL™ DNA Fragmentation Detection Kit (Merck, Germany) according to the manufacturer’s recommendation. For quantification, three different fields were counted under light microscopy and at least 500 cells were enumerated in each field. All experiments were performed in triplicate.

### APL murine model

Murine xenograft APL model was established by NB4 cells inoculation in nude mice. Briefly, mice were pretreated with 3 Gy of total body irradiation, which is a sublethal dose that was expected to enhance the acceptance of xenografts. Subsequently, NB4 cells (1 × 10^7^) were inoculated subcutaneously into the right flank of nude mice (male, 5–6 weeks of age). Inoculated NB4 cells formed subcutaneous tumors at the injection site from 6–8 days. Ten days after cell inoculation, mice were randomly divided into two groups, and received water (n = 5) or Catechins (10 mM, n = 5) as the sole drinking treated for 21 days. Tumor volumes were calculated by the formula: 0.5 × a × b2 in millimeters, where ‘a’ is the length and ‘b’ is the width. Tissue samples were fixed in formaldehyde and further embedded in paraffin.

### Statistical analysis

All the results were expressed as the mean ± S.D. and determined using t-test to compare variance. Survival functions were estimated using the Kaplan-Meier method and compared by the log-rank test. P value < 0.05 were considered statistically significant. All statistical analyses were evaluated using the SPSS for Windows, Version 18.0.

## Abbreviations

APL: Acute promyelocytic leukemia
PML: Promyelocytic leukemia
ATRA: All-trans retinoic acid
As_2_O_3_: Arsenic trioxide
EGCG: Epipallocatechin gallate
EC: Epicatechin
ECG: Epicatechin gallate
EGC: Epigallocatechin
MTT assays: 3-[4,5-dimethylthiazol-2-yl]-2,5 diphenyl tetrazolium bromide assays
IC50: The half maximal inhibitory concentration
Δψm: Mitochondrial trans-membrane potential
ROS: Reactive oxygen species
TUNEL: Terminal deoxytransferase-catalyzed DNA nick-end labeling assay
